# Old genes in new places: A taxon-rich analysis of interdomain lateral gene transfer events

**DOI:** 10.1371/journal.pgen.1010239

**Published:** 2022-06-22

**Authors:** Auden Cote-L’Heureux, Xyrus X. Maurer-Alcalá, Laura A. Katz

**Affiliations:** 1 Department of Biological Sciences, Smith College, Northampton, Massachusetts, United States of America; 2 Institute of Cell Biology, University of Bern, Bern, Switzerland; 3 Program in Organismic Biology and Evolution, University of Massachusetts Amherst, Amherst, Massachusetts, United States of America; Cornell University, UNITED STATES

## Abstract

Vertical inheritance is foundational to Darwinian evolution, but fails to explain major innovations such as the rapid spread of antibiotic resistance among bacteria and the origin of photosynthesis in eukaryotes. While lateral gene transfer (LGT) is recognized as an evolutionary force in prokaryotes, the role of LGT in eukaryotic evolution is less clear. With the exception of the transfer of genes from organelles to the nucleus, a process termed endosymbiotic gene transfer (EGT), the extent of interdomain transfer from prokaryotes to eukaryotes is highly debated. A common critique of studies of interdomain LGT is the reliance on the topology of single-gene trees that attempt to estimate more than one billion years of evolution. We take a more conservative approach by identifying cases in which a single clade of eukaryotes is found in an otherwise prokaryotic gene tree (i.e. exclusive presence). Starting with a taxon-rich dataset of over 13,600 gene families and passing data through several rounds of curation, we identify and categorize the function of 306 interdomain LGT events into diverse eukaryotes, including 189 putative EGTs, 52 LGTs into Opisthokonta (i.e. animals, fungi and their microbial relatives), and 42 LGTs nearly exclusive to anaerobic eukaryotes. To assess differential gene loss as an explanation for exclusive presence, we compare branch lengths within each LGT tree to a set of vertically-inherited genes subsampled to mimic gene loss (i.e. with the same taxonomic sampling) and consistently find shorter relative distance between eukaryotes and prokaryotes in LGT trees, a pattern inconsistent with gene loss. Our methods provide a framework for future studies of interdomain LGT and move the field closer to an understanding of how best to model the evolutionary history of eukaryotes.

## Author summary

Typical models of evolutionary biology focus exclusively on how genetic material is passed on “vertically” from parents to their offspring through reproduction. However, there is another kind of inheritance that is not so clear-cut but which has important effects on many groups of organisms. The “lateral” transfer of genetic material, transfer of genetic material between individuals that is not from parent to offspring, profoundly impacts the evolution of bacteria and archaea (i.e. prokaryotes). Rapid resistance to antibiotics is largely driven by this well-accepted phenomenon, and the rise of broad-scale genomic datasets has allowed detailed exploration of lateral gene transfer events in both prokaryotes and eukaryotes. However, there are few widely accepted standards for detecting genes that have been laterally transferred in eukaryotes, where many lineages remain under sampled. Here, we use a taxon-rich phylogenomic approach to develop and deploy a suite of methods for analyzing lateral gene transfer in eukaryotes. We first identify several hundred genes that have likely been transferred laterally from prokaryotes to eukaryotes, then we characterize their functions to detect broad-scale patterns, and finally, for most of these events, we reject the alternative hypothesis: gene loss explains the topology of laterally-transferred gene trees. Together, these data and approaches will be of use to future studies of LGT events in lineages across the eukaryotic tree of life.

## Introduction

Lateral gene transfer (LGT), the transfer of genetic material that is not from parent to offspring, is often neglected in models of eukaryotic evolution. This is problematic given the potential innovations enabled by such events and in light of existing data on the ubiquitous nature of LGT in bacteria and archaea. In some archaeal lineages, for instance, LGT is so pervasive that linkage disequilibrium is near that of sexual eukaryotes [1], and LGT is a major driver behind phenomena such as antibiotic resistance in bacteria [2,3]. Although first discovered and most widely studied in bacteria, LGT is not strictly limited to prokaryotes; endosymbiotic gene transfer (EGT), the transfer of genes from mitochondrial or plastid genomes (or endosymbiont nuclei/nucleomorphs in photosynthetic eukaryotes that acquired plastids from other eukaryotes) to the nucleus, is also well-documented [4–6]. Outside of EGT, however, the extent to which LGT affects eukaryotic evolution is debated [7,8]. Some have argued that, while EGTs are relatively common, other interdomain transfer events are very rare and have little effect on eukaryotic genomes [4]. Other studies suggest LGTs are not so uncommon [8–15], though the preponderance of gene loss has likely obscured these events [16].

Past attempts to identify laterally transferred genes (LTGs) in eukaryotes have relied on detecting deviations from eukaryotic monophyly in single-gene trees and, to a lesser extent, discoveries of aberrant composition to identify very recent (i.e. not yet ameliorated) LTGs, though due to rapid amelioration many past studies have found no significant difference in GC content between putative LGTs and other protein-coding genes [17–22]. We apply filtration by composition in a phylogenomic context to mitigate the effect of contamination in transcriptomic data. Sequence-similarity and BLAST-based metrics such as the alienicity index have been used to detect LGTs in eukaryotes, though some have argued that these methods are best as a starting place for selecting candidate genes before proceeding with more detailed analyses. The finding of numerous spurious LGTs in the human genome is a notable example of problems with relying too heavily on approaches such as these [22–27].

In many situations, detecting deviations from eukaryotic monophyly (i.e. a subset of eukaryotes appear to fall nested among prokaryotes, though other eukaryotes possess the gene) is challenging in that it requires estimating single gene trees at large time scales, and such topologies are often also consistent with other evolutionary scenarios such as ancient paralogy and subsequent differential gene loss [4,28]. Furthermore, assessment of tree topologies using likelihood-ratio tests frequently fail to reject eukaryotic monophyly, demonstrating the lack of support in these single-gene trees [29]. Indeed, some LTGs identified based on aberrant gene-tree topologies have turned out to be spurious—results of contamination or lack of data from other eukaryotic lineages [23,30]. In recent years, a number of studies have developed and applied methods to combat these pitfalls, including detailed analysis of sequence divergence between putative donors and recipients relative to outgroup eukaryotes [31–36].

Here, we present an approach for detecting LGTs from prokaryotes to eukaryotes that captures the evolutionary history and functional landscape of these events among diverse lineages. We rely on PhyloToL, a phylogenomic pipeline developed by our group, coupled with a taxon-rich dataset that prioritizes whole genome sequences and then transcriptomic datasets to encompass the diversity of eukaryotes [16,37]. Then, rather than attempt to interpret the topology of gene trees that contain disparate groups of eukaryotes, we focus on gene families (GFs) present only in prokaryotes and a specific “recipient” eukaryotic group to the exclusion of other eukaryotes (i.e. exclusive presence). We identify candidate LTGs only after intensive data curation (i.e. assessment of contamination in transcriptomic data, and the evaluation of individual scaffolds of whole genome taxa) and thorough exploration of alternative hypotheses, and show the efficacy of using rigorous criteria for identifying candidate LTGs.

In total, we analyzed whole genome and transcriptome data from 1,531 species (genomic data from 688 bacteria, 114 archaea, and 189 eukaryotes, plus an additional 540 eukaryotes with transcriptomic data) accessed from GenBank or generated by our lab (S1 Table). We mitigated contamination by removing low quality and highly contaminated transcriptomes, as well as sequences with aberrant composition as compared to a set of conserved gene families (see methods). For taxa with genome sequence data, we further curated data by mapping sequences of interest to genomic scaffolds and analyzing the nearby protein-coding regions (CDS), only accepting LTGs located on scaffolds longer than 10 kb for which we could identify nearby CDSs with BLAST hits to closely-related eukaryotes. To mitigate the possibility of scaffolding errors due to incorporation of bacterial contamination (especially in genomes assembled from short-read data), we carefully analyzed coding regions in the vicinity of each gene of interest, giving special manual attention (e.g. by extensive sequence similarity searching against prokaryote genomes) to regions with several nearby putative LTGs (S9 and S10 Tables). Through a combined analysis of Gene Ontology [38] (GO) terms and PFam [39] domains, functional characterization of the LTGs revealed trends in the functional distributions both within and between recipient categories.

## Results and discussion

### Analysis of interdomain LGTs in eukaryotes

**Fig 1:**
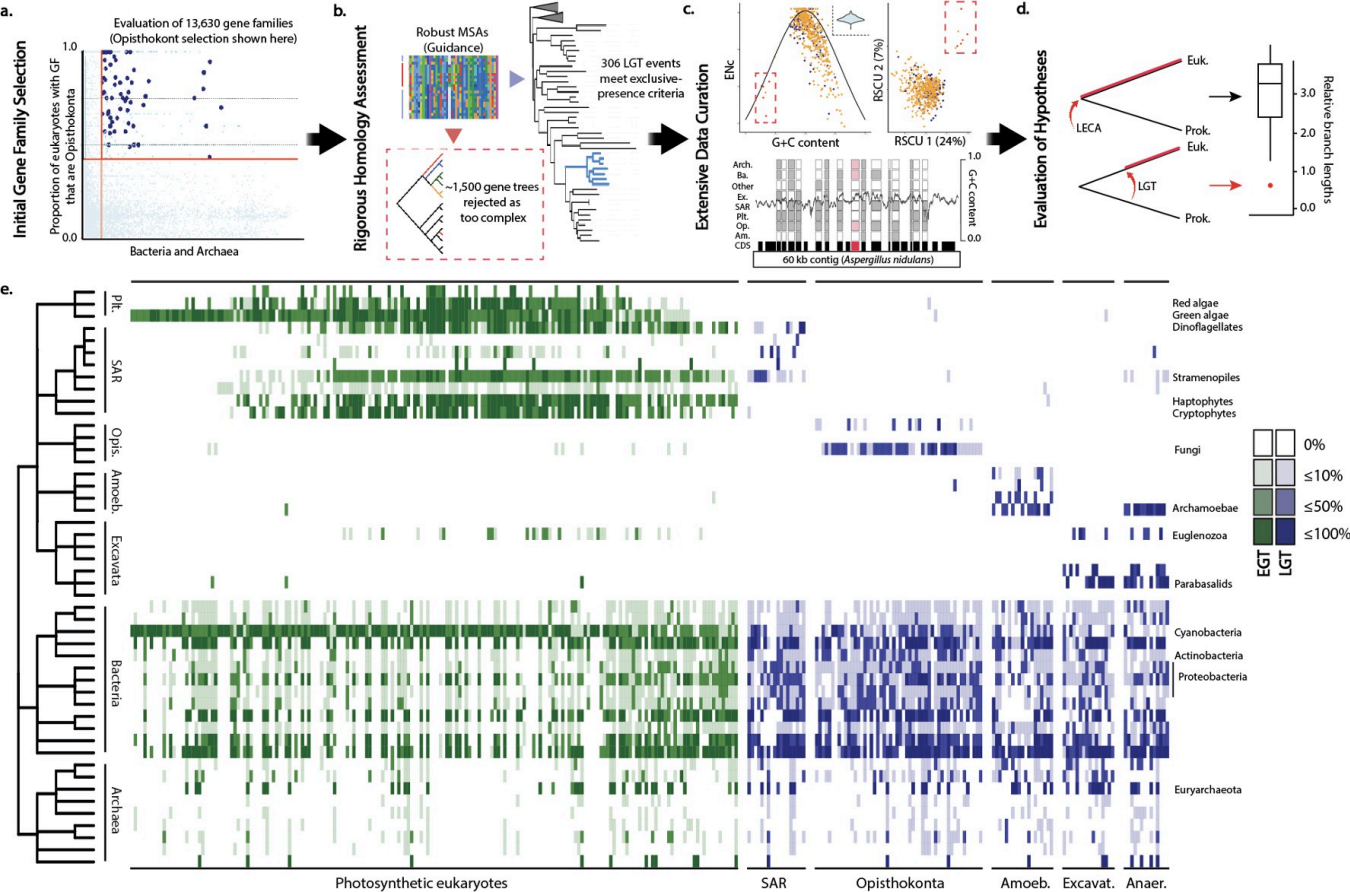
Rigorous methodology for LGT identification, curation and analysis uncovers 306 interdomain LGT events. (A) Using our taxon-rich phylogenomic pipeline PhyloToL, we initially identified 1,738 gene families as potential interdomain EGT/LGTs based on both the proportion of eukaryotes initially assigned to each GF that are of the target recipient clade (y axis) and number of prokaryotic sequences (x axis). (B) We used Guidance to assess homology and evaluated tree topologies for all candidate LTGs. (C) We curated sequences by analyzing patterns of compositional bias and codon usage for transcriptomic data (top) and by retaining only robustly-mapped sequences for genomic data (bottom). (D) To test the alternative hypothesis of differential gene loss, we analyzed relative branch lengths between putative recipients and donors. (E) Candidate LTGs exhibit exclusive presence in prokaryotes and ‘recipient’ eukaryotes; each column represents an interdomain transfer event. Eukaryotes (top) are shaded by the proportion of the taxa in the subclade (row) that appear in the tree, and prokaryotes (bottom) are shaded by the proportion of prokaryotes in the tree that are of the subclade. Abbreviations are as follows: Plt: Archaeplastida; SAR: Stramenopila, Alveolata, and Rhizaria; Opis: Opisthokonta; Amoeb: Amoebozoa; Excavat: Excavata; Anaer: Anaerobes.

We identified 306 gene transfer events from 295 GFs into a variety of eukaryotic clades (S2 and S3 Tables). Many of these are instances of putative endosymbiotic gene transfer (EGT), which we consider separately from other LGT events. Using the phylogenomic pipeline PhyloToL [40], the initial selection of GFs based on the presence of potential recipients yielded over 1,700 candidate LTGs (Fig 1A). We then generated multi-sequence alignments for these GFs using Guidance v. 2.02 [41], a tool that allows rigorous homology assessment, and then constructed gene trees using RaxML v. 8.0 [42] as incorporated into PhyloToL (Fig 1B). After extensive data curation, including visual inspection of all alignments and trees, we retained only LTGs for which all or nearly all of the eukaryotes belong to a targeted recipient clade or group (Fig 1C). This approach is conservative in that it will exclude ancient events, cases where a vertically transmitted gene was lost and then re-acquired from a prokaryote, and most cases of intradomain (i.e. eukaryote-to-eukaryote) transfer (e.g. [43]).

We find certain prokaryotic groups overrepresented in distinct categories of LTGs, though discerning donor lineages in individual LGT events is confounded by a variety of factors including rampant gene transfer among prokaryotes [3,29,44–46]. The case of Cyanobacteria as the endosymbiont ancestor in EGT trees is the most prominent (Figs 1E and S4). Our approach selected against EGTs from the mitochondrial genome as these transfer events tend to be very ancient [47] and therefore generally do not show exclusive presence. Regardless, we do not see a disproportionate amount of Alpha-proteobacteria (the mitochondrial endosymbiotic ancestor) relative to other bacterial groups in putative LTG trees (Figs 1E and S4). In contrast, a recent study found enriched proteobacterial presence in putative LTGs to phytoplankton, which was explained by the ecological similarities of the putative donor and recipient lineages [48].

While differential gene loss remains a valid explanation for how exclusive presence can arise [4,28], this would lead to the expectation of relatively large divergence between eukaryotes and prokaryotes in LTG trees. To assess whether LTGs were more recently in prokaryotic ancestors than vertically-transmitted genes (VTGs; Fig 1D), we conducted a test based on branch-lengths to identify systematic biases, similar to analyses that have been proposed and/or conducted in recent studies of LGT, though these studies were conducted without the explicit intent of comparing to simulated instances of gene loss [31,33,49]. We first selected a group of putative VTGs based on their presence in all five major clades of eukaryotes (see methods). Next, we mimicked gene loss by subsampling these VTG trees to match the taxonomic distribution of both eukaryotes and prokaryotes for a given LTG. We then generated an alignment and gene tree for each subsampled VTG to compare the ratios of the average branch length within the eukaryotic clades to the distance between the eukaryotic clade and the last common ancestor of the prokaryotes.

**Fig 2:**
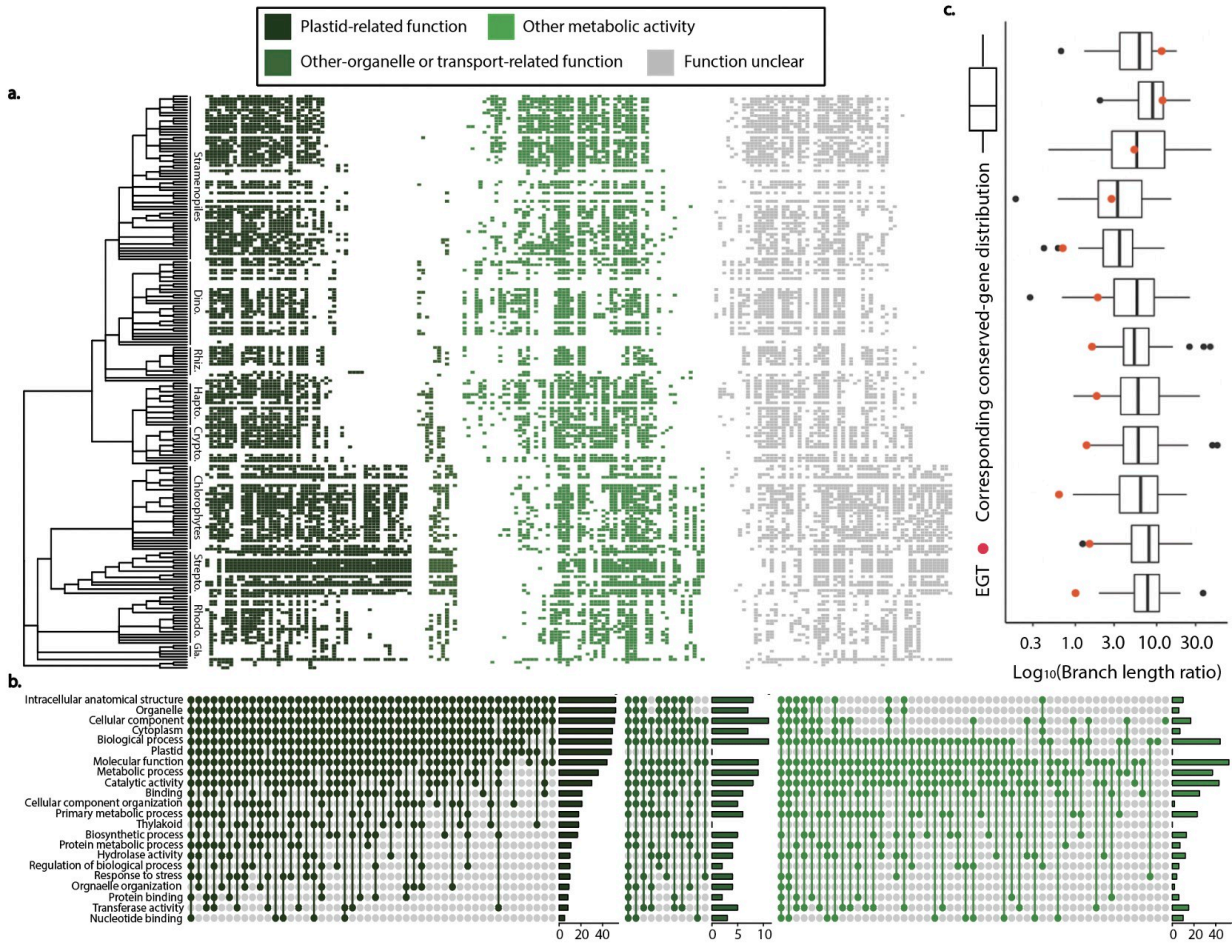
The taxonomic and functional distributions of putative EGTs. (A) The distribution of 189 GFs, categorized by putative function, subject to transfer events (columns) across all photosynthetic eukaryotes included in the study (Dino: Dinoflagellates; Rhiz: Chlorarachniophytes (Rhizaria); Hapto: Haptophytes; Crypto: Cryptophytes; Strepto: Streptophytes; Rhodo: Rhodophytes; Gla: Glaucophytes). (B) Functional categories based on the co-occurrence of Gene Ontology terms designate 52 EGTs as plastid-related (dark green, lefthand panel), 10 as metabolic, catabolic or biosynthetic (medium green, central panel), and 61 as other organellar or transport related (light green, right panel). (C) In comparisons of relative branch lengths, putative EGTs into Archaeplastida (red dots) consistently fall below the relative distance between eukaryotes and prokaryotes in VTG trees (box plots), inconsistent with the alternative hypothesis of gene loss and consistent with the results of similar analyses in other, non-photosynthetic taxonomic groups such as Opisthokonta (Fig 3).

**Fig 3:**
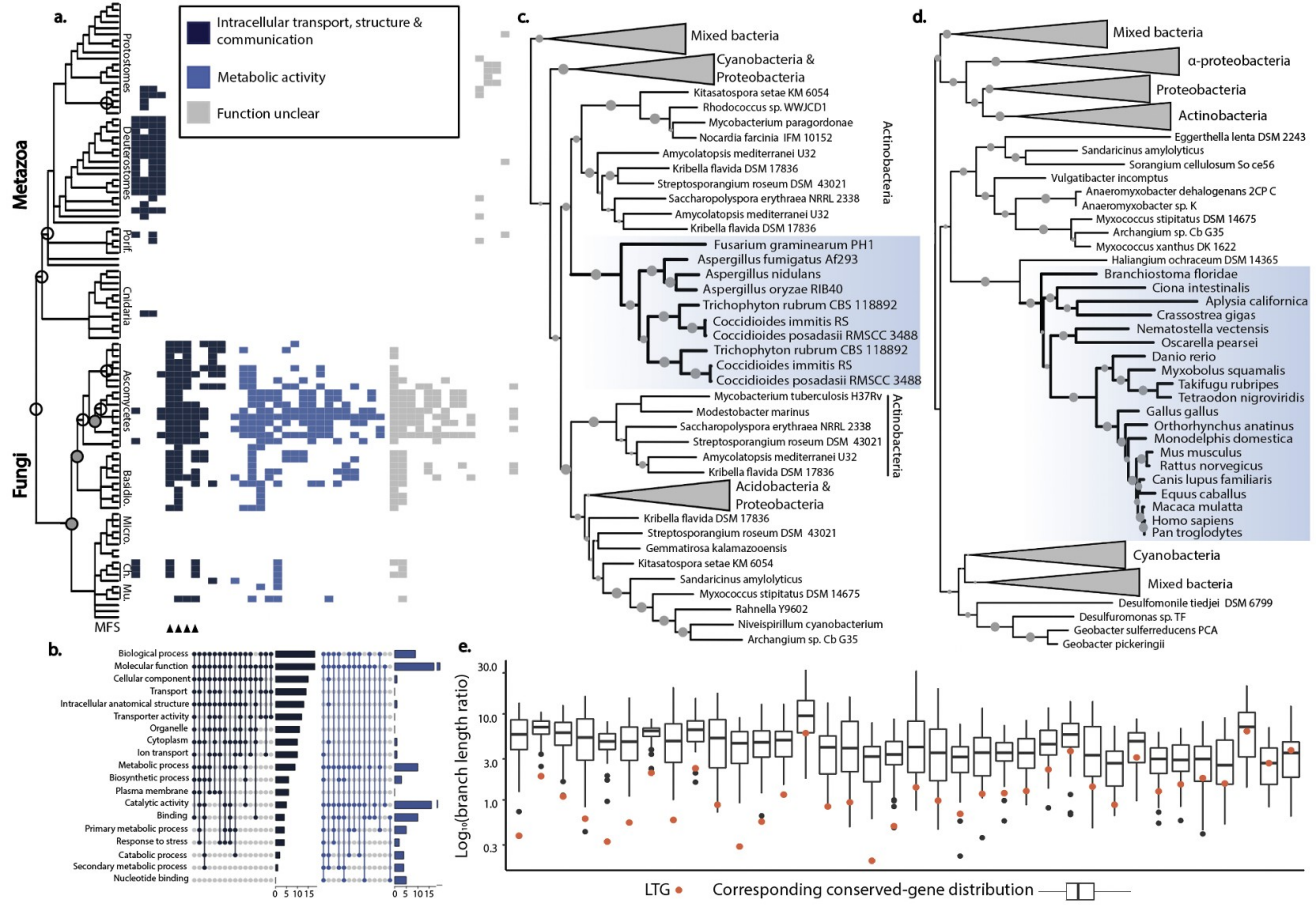
A summary of 52 interdomain LGT events in Opisthokonta, most of which are unique to fungi. (A) The presence of GFs (columns) in opisthokont species, categorized by function (colors). Nodes with fewer than five and five or more inferred events are represented by open and closed circles, respectively. The presence of the MFS_1 domain is indicated by triangles. (B) Gene families categorized by function using Gene Ontology terms fall into two major categories: intracellular transport, structure and communication (dark blue) and metabolic activity (light blue). (C) Exemplar trees showing LGT into fungi and (D) Metazoa with bootstrap values greater than 50% denoted by the gray circles. Blue boxes indicate all eukaryotes (Opisthokonta) in the tree. (E) In relative branch length comparisons, Opisthokont LTGs (red dots) consistently fall below the first quartile of the relative distance between eukaryotes and prokaryotes in VTG trees (box plots), inconsistent with the alternative hypothesis of gene loss.

**Fig 4:**
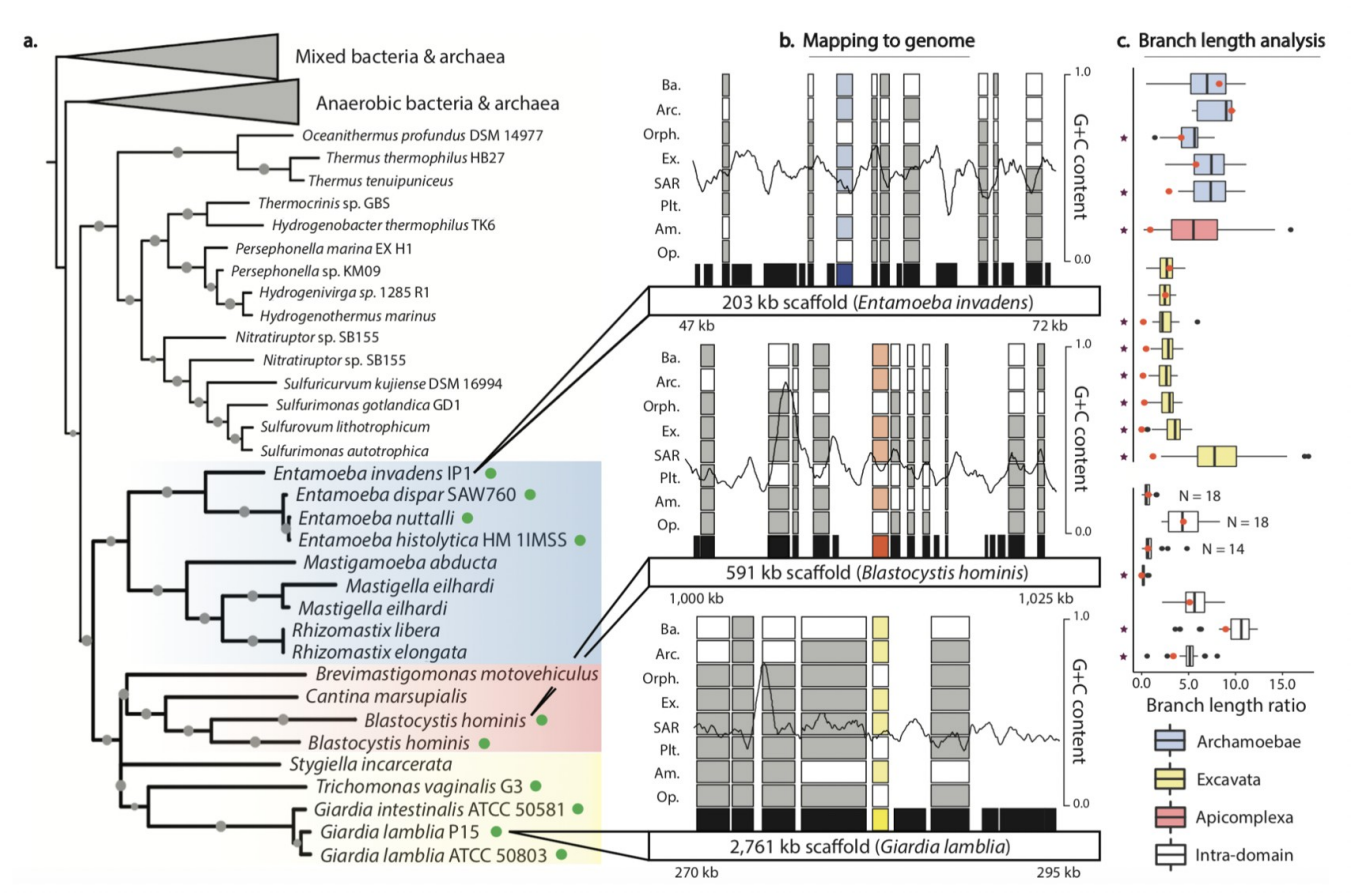
Using strict exclusive-presence criteria and extensive curation, we found evidence for both inter- and intradomain transfer involving anaerobic eukaryotes. (A) A glycyl-radical enzyme-activating enzyme (GRE-AE) is found exclusively in anaerobic eukaryotes (colored boxes) and prokaryotes. (B) Curation of genomic sequences (green dots in Fig 3A) kept only those that mapped to scaffolds longer than 10 kb and with robust nearby CDS as represented by the three scaffold sections: LTGs are shown in color and the presence/absence of nearby GFs in other eukaryotic and prokaryotic groups are shown by filled and empty squares, respectively (Ba: Bacteria; Arc: Archaea; Orphan: Eukaryotic orphan lineages; Ex: Excavata; Plt: Archaeplastida; Am: Amoebozoa; Op: Opisthokonta); sliding window GC content shows amelioration of these LTGs; CDSs (black boxes) with no presence/absence data are not in the PhyloToL database (e.g. lineage specific genes). (C) In most branch length comparisons, LTGs (red dots) fell below the first quartile of the branch-length ratios of the corresponding set of conserved genes (box plots) subsampled to mimic gene loss. This includes the distance between SAR and Excavata in the GRE-AE gene tree (A); the number of VTG trees is given for LTGs with fewer than 20 VTGs in their corresponding distribution.

Using this simple branch length comparison method, we found that the distance between the eukaryotes and prokaryotes in most LGTs was shorter than for the corresponding subsampled VTG, with the distance commonly falling below the first quartile of their corresponding VTG distribution (Figs 1D, 2C, 3E and 4C, and S11 and S12 Tables). Correct interpretation of the results is contingent on the homogeneity of substitution rates before and after transfer, and it is possible that some of the cases in which our LGTs match VGTs that mimic loss are due to elevated rates of evolution immediately following gene transfer. The low relative branch length ratios of some putative LGTs are driven by both a decreased distance between eukaryotes and prokaryotes (consistent with a hypothesis of LGT) and an increase in branch length among recipient eukaryotes (S1–S3 Figs), consistent with an accelerated rate of evolution post-transfer. These patterns are also consistent with elevated rates of evolution following gene loss in all but the remaining clade. Additionally, it is possible that some of these genes were transferred from eukaryotes into prokaryotes, but this is unlikely due to our selection criteria of exclusive presence; in our candidate LTGs, the relative diversity of prokaryotes is almost always much greater than that of eukaryotes.

We identified putative LTGs in either monophyletic eukaryotic groups or into groups of organisms that share emergent functional properties (i.e. photosynthetic or anaerobic lineages). In total, we found 189 putative EGTs, 52 LGT events in Opisthokonta, 19 in Amoebozoa, 16 within Excavata, and 17 among SAR (Stramenopila, Alveolata, and Rhizaria); we also identified 14 LTGs unique to anaerobic eukaryotes belonging to two or more major eukaryotic groups, which we hypothesize may involve intra-domain transfer. We created a separate pipeline to assess the potential function of relatively ancient (and, by our methods of detection, widely retained) LTGs in eukaryotes. Numerous studies have explored the functions of individual putative LTGs on a case-by-case basis. For example, an analysis of the genome of the choanoflagellate *Monosiga brevicolis* revealed hundreds of putative LTGs, the majority of which are involved in carbohydrate and amino acid metabolism or stress responses [50], and another study of bacterial transfer into Ochrophyta emphasized the possible role of LGT in the evolution of secondary metabolic pathways in eukaryotes [51]. Our methods of LTG identification and curation allowed us analyze the high-level functional trends of LTGs in each taxonomic group; below we discuss these cases in three sections: 1) EGTs; 2) LGTs into non-photosynthetic eukaryotes; and 3) the special case of LGT into and between anaerobic eukaryotes.

### EGTs into photosynthetic eukaryotes

Endosymbiotic gene transfer is distinct from other forms of non-vertical gene transfer in that its mechanism is relatively well understood, and it results from transfer of genes from a permanent intracellular symbiont [5,6]. As transfer of genes from endosymbionts to the nucleus is well-documented in photosynthetic eukaryotes [4–6,52–55], we use EGTs as a control for our assessment of other interdomain gene transfers and as a pilot for our deployment of a functional analysis pipeline. We characterize gene families as EGTs when they are exclusively or nearly-exclusively found in prokaryotes and photosynthetic eukaryotes. Photosynthetic lineages in our pipeline include Archaeplastida (green algae, red algae, glaucocystophytes) whose ancestor acquired a plastid from a cyanobacterium [56], and clades that acquired plastids from other eukaryotes through secondary or tertiary endosymbiosis (eg. many stramenopiles, dinoflagellates, haptophytes, and cryptophytes) [52,57]. We consider genes present in only these clades as EGTs even if some lineages are thought to have lost the ability to photosynthesize, as there is a strong possibility that genes were transferred into the nuclear genome before ancestral plastid loss. Another alternative is that some of these events are transfers from the nucleus (or nucleomorph) of a photosynthetic eukaryote, with only some of these genes being originally of cyanobacterial origin.

Given the complex evolution of genes involved in photosynthesis, for which numerous hypotheses have been proposed [57,58], we recognize that our simplistic approach may categorize some LGTs as EGTs; any genes transferred laterally from a free-living prokaryote (i.e. not an endosymbiont) into a photosynthetic lineage will be incorrectly categorized by our study. While this is a possibility, it would be difficult to confidently rule out EGT partly because frequent LGT and gene loss among prokaryotes can obscure inferences on possible donors. As EGTs appear to be more common and stable than other forms of non-vertical transfer, we chose EGT as the most parsimonious hypothesis. Another scenario that we consider EGT is if a gene were to be transferred from a free-living bacterium into a eukaryote that later became an endosymbiont, and in turn transferred from the degrading nucleomorph into the recipient genome.

As expected, more Cyanobacteria appear in EGT trees than in any other category of LTGs (Figs 1E and S4), consistent with the cyanobacterial ancestry of the plastid [56,59]. We also find topologies consistent with the secondary and tertiary transfers of plastids, as lineages of photosynthetic eukaryotes frequently nest among archaeplastida; our trees also include lineages that have likely lost their plastid but retained genes of plastid-origin in their nuclear genome (e.g. Apicomplexa and Perkinsozoa, which appear in many of our EGT trees; S6 Fig) [52,58,60–62].

Deploying a pipeline that automates assessment of function through analysis of GO terms and PFam domains (see methods), we find that EGTs are frequently assigned plastid-related functions (Fig 2), consistent with previous literature [4,5,63]. Most of the EGTs exclusive to photosynthetic eukaryotes that acquired their plastid secondarily were assigned metabolic, catabolic or other biosynthetic functions (Fig 2). These candidate EGTs have elevated levels of Alpha-proteobacterial presence relative to other EGTs and are less likely to have a plastid-related function than trees containing Archaeplastida (Fig 2), consistent with plastid-related genes of non-cyanobacterial origin, a phenomenon which has been documented [57,58,60,64]. Our approach to the functional analysis of EGTs contrasts with the more ‘piecemeal’ approach of previous studies in which researchers focus on exploration of one or a few candidate genes or lineages [53,65–69]. Also, many studies focus either on functional differences between genes retained in the plastid as compared to those that are transferred to the nucleus, or on EGTs in lineages that have subsequently lost the ability to photosynthesize [55,70]. When the more general functional distribution of putative EGTs has been thoroughly analyzed and compared to other groups of gene families, it has been with a relatively small number of photosynthetic eukaryotes [4]. In contrast, we use EGTs to exemplify a functional analysis pipeline that, combined with intensive manual data curation, takes full advantage of our taxon-rich dataset to make inferences on the function of genes in the recipients of EGT (Fig 2A and 2B).

We also assess the alternative hypothesis of gene loss as an explanation for the 12 putative ETGs that are only present in Archaeplastida and bacteria (and sometimes only cyanobacteria), and that have taxonomic distributions that match (subsampled) conserved gene trees. Notably, the relative distance between eukaryotes and prokaryotes was below the first quartile of that of the VTGs in 8 out of 12 cases, and above the median in only two (Fig 2C), a trend inconsistent with gene loss and consistent with EGT. Moreover, the trends in the distance between eukaryotes and prokaryotes in these EGTs, the average branch length within eukaryotes, and their ratio, are similar to those of LTGs found exclusively in non-photosynthetic eukaryotes (S1–S3 Figs).

### LTGs unique to non-photosynthetic eukaryotes

Using the requirement of exclusive presence, we identify a total of 52 interdomain LGT events into Opisthokonta: four into a common ancestor of all Opisthokonta, 41 specific to fungi, and seven specific to metazoa (Fig 2A). Within fungi, we found 28 LGTs specific to Dikarya, with 9 transfers unique to ascomycetes and no transfers solely into Basidiomycetes despite the presence of 10 Basidiomycetes with completed genomes in our database. While Chytridiomycetes and Mucoromycetes are recipients of several LGTs, we found no LTGs retained in the genomes of microsporidia (a causative agent of wasting diarrhea in patients with AIDS), which likely reflects both elevated rates of gene loss in these highly-streamlined genomes [68,71] as well as our limited sampling of the group and focus on ‘older’ events, as LGTs have previously been documented in this lineage [72]. We did discover several putatively recent transfer events unique to single fungal species when assessing candidate LTG into other lineages. For example, we detect a transfer of an EPSP synthase (OG5_131267) gene from Proteobacteria into *Aspergillus oryzae* in a gene tree that we first identified for the presence of green algae nested among Cyanobacteria. As with other such cases, our curation mapped this gene to a robust genomic scaffold, found no other BLAST hit to eukaryotes, and rejected the monophyly of the eukaryotes on this tree by AU test, indicating multiple putative transfer events for this gene family (S2 Table).

The majority of the GFs putatively transferred into Opisthokonta have functions related to either: 1) intracellular transport, structure, and communication; or 2) metabolic activity (Fig 3C and 3D). Though the GFs belonging to the first category are found in both metazoa and fungi, LTGs involved in metabolic and biosynthetic function are unique to fungi. Additionally, four of the five ‘intracellular transport’ LTGs transferred into the last common ancestor of fungi contain the major facilitator superfamily (MFS_1) domain (Fig 3A), expanding on previous work suggesting that some MFS subfamilies originated from a limited number of transfer events into fungi [73].

Across the 39 GFs putatively transferred into Opisthokonta that met our criteria for branch-length comparison, only six are consistent with a scenario of gene loss: one LTG lies above the median of its corresponding VTG distribution, and five fall above the first quartile. These six LTGs that fall above the first quartile represent either cases of gene loss that give rise to trees with exclusive presence or cases in which changes in functional constraints altered relative branch lengths (i.e. a rapid period of protein evolution as a transferred gene is first incorporated into the recipient genome). For the remaining 33 GFs that lie below the first quartile, 12 fall entirely outside of the estimates for vertical trees that mimic gene loss (Fig 2E). This pattern, which is consistent with LGT and not gene loss, is as or more pronounced as in cases of putative EGT (Fig 2C).

The presence of Actinobacteria is substantially greater in GFs putatively transferred into fungi (Figs 1E and S4), consistent with literature proposing early transfer events from Actinobacteria into the ancestor of fungi [74]. The striking similarities between fungi and Actinobacteria, from morphology to shared environment, have been linked to the potentially important role of ecology in determining patterns of transfer [75,76]. The potential role of shared ecology is further demonstrated by evidence for gene transfer between fungi and oomycetes [77]; though we focus on interdomain events, we recover a putative decarboxylase with a gene tree topology and presence/absence pattern consistent with lateral transfer from bacteria to fungi followed by transfer from fungi to *Phytophthora ramorum*, the sudden oak death pathogen.

Lateral transfer has been extensively studied in Opisthokonta, especially in fungi, where intimate symbiotic relationships (i.e. in lichens and mycorrhizal species) that often involve prokaryotes have predisposed some lineages to higher rates of LGT [35,78–82]. In fact, interdomain LGT has been implicated in important fungal innovations from gravity-sensing organs to pathogenic mechanisms and toxin-encoding genes [34,35,83,84]. In addition, some fungal species and genera seem to be especially prone to receiving LGTs, including the genera *Aspergillus* and *Fusarium*, for which genomic data are available [35,85] and in which we also observe frequently in putative LTG trees. LGT into other opisthokonts has been a point of interest to many, especially since the early spurious claims of hundreds of LTGs in the human genome [25,26], but specific mechanisms underlying individual transfers remain unknown. In fungi, *in vitro* experiments have been able to introduce foreign genetic material from prokaryotes into various fungi [86]. Proposed mechanisms for LGT *in vivo* include transfer mediated by mobile genetic elements (e.g. viruses or transposable elements) and/or during long-term associations with parasites or symbionts [15,87].

In addition to Opisthokonta, we identified LTGs specific to one of three other major eukaryotic clades: 19 to Amoebozoa, 16 within Excavata, and 17 to SAR (Stramenopila, Alveolata, and Rhizaria). Many of the LTGs in the former two groups are found in anaerobic obligate parasites (e.g. *Entamoeba* (Amoebozoa), *Trichomonas* and *Giardia* (Excavata); S3 Table), which likely reflects both bias in the available data and also possibly gene transfers that support a transition to a strictly anaerobic lifestyle (see “LGT into anaerobic eukaryotes” below). Within Amoebozoa, we recover multiple putative LTGs in the slime mold *Dictyostelium*, such as a previously reported [88] siderophore transport-related protein (important in iron scavenging), plus other undocumented transfers into Discosea, Mycetozoa, and Tubulinea (S2 Table). The LTGs exclusive to members of SAR are found primarily in oomycetes and other stramenopiles (N = 11), apicomplexans (N = 4), and ciliates (N = 3). Apicomplexans such as *Plasmodium* also occasionally appear sister to photosynthetic eukaryotes in EGT trees, consistent with the presence of the plastid-derived apicoplast in these lineages (S6 Fig). Though our findings are generally consistent with the numerous studies that report interdomain LTGs into the predominantly microbial lineages of Excavata, Amoebozoa, and SAR, our stringent methods contrast with analyses that focus on specific taxa and/or rely on topologies of single gene trees [22,70,77–79].

### LGT into anaerobic eukaryotes

Multiple transitions from aerobic to strictly anaerobic/microaerophilic life strategies required eukaryotes to alter fundamental features of their metabolism, and previous analyses suggest that this involved the acquisition of genes from prokaryotes [10,12,89–93]. Such transfers are analogous to the emergence of photosynthesis in eukaryotes after the acquisition of the plastid through endosymbiosis, which led to a combination of inter- and intradomain EGTs. Endosymbioses and other intimate relationships among anaerobic lineages may also provide opportunities for transfers; consistent with the hypothesis is the presence of the anaerobic endosymbiont *Perkinsella* (Excavata) within the amoebozoan *Paramoeba* [94].

Given that the last eukaryotic common ancestor (LECA) likely contained pathways for both aerobic and anaerobic metabolism [95,96], interpreting the evolutionary history of ‘anaerobic’ genes must be done with caution. Indeed, some past findings of putative LTGs specific to anaerobes have been revised with additional data from homologous pathways in aerobic lineages [95]. For example, the sparse distributions among paraphyletic eukaryotes of genes involved in the anaerobic conversion of pyruvate to Acetyl-CoA, such as pyruvate-formate lyase (PFL), its activating enzyme (PFL-AE), and pyruvate:ferredoxin oxidoreductase (PFO) have led to the suggestion of interdomain followed by intradomain transfer [10,89,97]. In contrast to this hypothesis, the presence of these genes in various aerobic lineages (e.g. green algae) suggests that they were present in LECA and underwent extensive differential loss [95].

We identified 42 GFs found exclusively or nearly exclusively in eukaryotes sharing anaerobic functional and/or ecological contexts (e.g. Archamoeba, Parabasalids, Trypanosomatids, and Apicomplexa); in many of these cases, the eukaryotes are sister to anaerobic and/or pathogenic bacteria (S3 Table). Several previously reported anaerobic LTGs did not pass our conservative criteria due to their widespread presence in other eukaryotes, including PFL, PFL-AE, and PFO [10,89,97,98]. However, we did recover a glycyl-radical enzyme-activating enzyme (GRE-AE) highly similar to PFL-AE that was previously documented in *Giardia* [12], and later *Entamoeba* and *Trichomonas* [99]; we additionally find it in other Archamoebae and anaerobic members of SAR (Fig 4A). We expand the taxonomic scope of other previously-reported GFs, such as the alcohol dehydrogenase EhADH3B originally reported as an LTG unique to the human parasite *Entamoeba histolytica* [100] where it is associated with pathogenicity [101]; we recovered this GF in other Archamoebae, *Trichomonas*, and *Blastocystis*. Similarly, we find the nitroreductase Fd-NR2 in *Entamoeba*, expanding its presence from a previous report as an LTG only in *Giardia* [102] (S3 Table).

To further assess candidate anaerobe LTGs, we carefully curated each sequence by mapping it to the genome to account for contamination (Fig 4B and S9 and S10 Tables) and tested the alternative hypothesis of gene loss using relative branch length comparisons (Fig 4C). We only retained sequences that mapped to scaffolds longer than 10 kb and with clearly eukaryotic coding domains on the same scaffold as the LTG (Fig 4C). We excluded from our study several putative LTGs in the excavate *Trimastix marina* and in the breviates *Pygsuia biforma* and *Lenisia limosa*, as these transcriptomes contained evidence of contamination by other eukaryotes in our analyses of control groups of genes conserved across eukaryotes (S9 and S10 Tables). Of the 14 interdomain LGTs that met our criteria for relative branch-length comparison, the majority lay below the first quartile and four are above the median (i.e. consistent with gene loss; Fig 4C), inconsistent with gene loss as an explanation for the pattern of inheritance of most of these putative LTGs. Fourteen anaerobe-specific LTGs are exclusive to paraphyletic eukaryotic lineages (i.e. contain anaerobic Amoebozoa, Excavata and/or SAR). Here we used the branch-length comparison approach to assess the seven cases of putative intradomain transfer that had large enough corresponding distributions of VTGs: three LTGs lay below the first quartile of the corresponding conserved-gene distribution and two lay above the median (Fig 3C), a signal consistent with LGT but with less support than the interdomain cases.

### Synthesis

Non-vertical inheritance of genetic material clearly confounds attempts to reconstruct the tree of life [3,103–106]. However, the extent to which this is a problem is difficult to discern with such varying standards as those currently used for discovering LGT, many of which leave differential gene loss as a possible alternative explanation [4,23,26,95]. Here we present rigorous and conservative methodology for identifying interdomain LGT events in eukaryotes: we require exclusive presence of GFs in taxon-rich analyses and apply multiple rounds of curation including analyzing compositional bias, mapping genomic cases to robust scaffolds, and carefully interpreting transcriptomes that often include sequences from contaminants (Fig 1A–1D). We identified 306 LGT events (Fig 1E) and then assessed the alternative hypothesis of differential gene loss to find that our candidate LTGs are consistently outliers (Figs 2C, 3E and 4C), though this varies between recipient groups. Using a definition based on exclusive presence and testing these hypotheses by branch-length ratio comparisons provides an important step towards clarifying criteria for robustly identifying LGT events and thus determining how pervasive LGT is as a phenomenon in eukaryotes.

## Methods

### Taxon selection

Analyses here rely on PhyloToL [40], which includes 540 eukaryotic species, 688 bacteria and 114 archaea, and represents a combination of whole genome taxa (all prokaryotes and 189 eukaryotes) plus numerous lineages represented by transcriptomes (i.e. ‘transcriptomic taxa’; Tables 1 and S1). Our intention in sampling was to create a relatively even set of taxa from across the eukaryotic tree of life, given data availability; to this end, we have undersampled plants, animals and fungi. Data are largely from GenBank (S1 Table), representing mostly lineages that can be cultivated. We also included 111 single cell transcriptomes from diverse microeukaryotes characterized in our lab that survived the rigorous data curation described below (S9 and S10 Tables). As part of data management, all taxa/cells are named with ten-digit codes that represent their major clade (e.g. Op = Opisthokonta),”minor” clade (e.g. Op_me = metazoa) and species (Op_me_Hsap = *Homo sapiens*). Though controversial, we include the major clade Excavata (Ex_), excepting the genus *Malawimonas*, and we note that the LGTs involving Excavata all include only a subset of the clade (i.e. do not rely on the monophyly of the group).

**Table 1:** The taxonomic breadth of data used in the study.

*Major clade*	*Constituent clades*	*# Genomes*	*# Transcript.*	*# LGTs*	*# EGTs*	*# Anaerobes*	*# Genera*	*# Species*
*Opisthokonta*	Fungi, Metazoa, Icthyosporea, Choanoflagellata	75	4	60	16	1	73	79
*Amoebozoa*	Archamoebae, Discosea, Tubulinea, Mycetozoa	8	59	35	3	8	43	67
*Archaeplastida*	Chlorophytes, Streptophytes, Rhodophytes, Glaucophytes	22	50	8	172	0	60	72
*SAR*	Stramenopila, Alveolata, Rhizaria	26	161	32	156	3	155	187
*Excavata*	Euglenozoa, Parabasalia, Heterolobosea, Fornicata, Jakobida, Oxymonada	18	9	35	32	21	18	27
*Other eukaryotes*	Cryptophytes, Haptophytes, Centroheliozoa	3	32	11	131	0	26	35
*Bacteria*	See S1 Table	653	0	120	189	NA	509	653
*Archaea*	See S1 Table	115	0	79	70	NA	86	115

### Initial curation of transcriptomic taxa

We evaluated all transcriptomic data prior to analysis of candidate LTG multi-sequence alignments (MSAs) and trees using PhyloToL [40] to assign transcripts to gene families. To mitigate both quality and contamination issues frequent with transcriptome data from microeukaryotes, we assessed the number of transcripts initially assigned to gene families (GFs) and the proportion of transcripts determined to be likely bacterial (i.e. contamination or potential LGTs), defined by PhyloToL as those that return a top BLAST hit to bacteria with an e-value at least 10^3^ times less than that of the top eukaryotic hit. We excluded samples with high ratios of putative contaminating bacterial sequences to non-bacterial sequences, and samples likely contaminated by food sources; this approach removed a total of 55 transcriptomes. We took a similar approach to identify eukaryotic transcriptomes contaminated by other eukaryotes based on a pilot analysis of 35 gene trees constructed from highly conserved gene families (i.e. those present in a large number of species across all seven major clades). We identified 253 transcriptomic samples (from our lab and GenBank) that either failed to appear in these trees (i.e. experimental failure) or that had high levels of contamination (e.g. by food source, commonly co-cultured lineages such as *Bodo*) assessed using our knowledge of the organisms’ ecology and data source. In a second round of taxon curation, we removed additional taxa and sequences (i.e. contaminants and/or sequences from food sources) based on their performance in sets of conserved genes (i.e. present across diverse eukaryotic lineages), including the 408 MSAs generated to explore compositional bias and codon usage (see below).

### Candidate gene-family selection

We started with 13,630 gene families (GFs) as defined by OrthoMCL [107] release 5.0 and incorporated them into PhyloToL, based on their presence in diverse eukaryotes (Table 1). For transcriptomes, PhyloToL translates nucleotide sequences ≥200bp after determining the appropriate genetic code and assigns amino acid sequences to GFs based on similarity using USEARCH [108] with a maximum e-value of 1e-10, and then combines these with sequences from OrthoMCL. We identified potential LTGs in predefined sets of potential recipient groups (Opisthokonta, Amoebozoa, Excavata, SAR, photosynthetic eukaryotes, and anaerobic eukaryotes) based on two relatively lenient criteria: 1) GFs where the proportion of the eukaryotes of the potential recipient clade were greater than the mean plus the standard deviation across all 13,630 GFs, and 2) the number of prokaryotes was greater than the 65th percentile across all GFs (Fig 1A). For cases where we had large numbers of single-cell transcriptome sequences (e.g. ciliates, Arcellinida), we applied a similar approach to sequences that PhyloToL determined to be possible bacterial contaminants (i.e. they had a BLASTx hit to a bacterial sequence in the OrthoMCL database that was at least 10^3^ times less than its best BLASTx hit to a eukaryote); here we chose GFs for which the proportion of taxa in a potential recipient clade fell significantly above the general distribution (identified using Mahalanobis distance; p-value < .001), with a minimum of at least 67% of the eukaryotes belonging to the recipient clade.

### Refinement of putative EGT/LGT gene families

We assessed homology for all candidate LTGs using up to five iterations of Guidance [41] version 2.02, a tool that removes sequences below user-specified thresholds (in our case, seqCutoff = 0.3 and colCutoff = 0.4) after multisequence alignment (MSA) reconstruction with MAFFT [109]. We masked gaps at 95% using trimAl [110] version 1.2 and constructed preliminary gene trees using RAxML [42] version 8.0 as implemented in PhyloToL (model PROTGAMMALG). We curated the resulting gene trees to focus on those that had nearly-exclusive presence of putative recipient clades (i.e. we allowed singletons or small second clades only if they survived our strict curation as described below) and then we went on to use several rounds of sequence curation described in the “Gene tree curation” section below. In other words, the gene trees were a tool for discovery of transferred gene families and we did not rely on single gene tree topologies as we finalized our list of LGTs.

We also curated all EGTs to avoid inclusion of plastid sequences present in transcriptomes. Using custom Python and R scripts, we compared putative EGT sequences against a set of 408 highly-conserved nuclear-encoded gene families (i.e. present in all five eukaryotic major clades) by plotting G+C content at third-position four-fold degenerate sites (GC3) against the effective number of codons (ENc) [111]. We also ran a correspondence analysis of the relative synonymous codon usage (RSCU) to distinguish nuclear and plastid genes. These analyses combined led to the removal of 29 putatively-plastid sequences that were significantly outlying (Mahalanobis distance; p-value < .001) in either of these plots (S5 Fig).

### Sequence-level curation of candidate LGTs

In addition to initial curation of taxa, we refined candidate LTGs to meet a set of conservative criteria. Because of bacterial by-catch in eukaryotic transcriptome studies, we removed clades of transcriptomic sequences with insufficient taxonomic representation. These clades are defined as containing only sequences designated by PhyloToL as likely bacterial (see above) that contained either <4 species or <33% of the single-cell transcriptomes generated in our lab for a given taxon. We also curated every sequence in gene trees that fell into any of four categories: 1) sequences sister to one or no other closely-related eukaryotes (e.g. an Amoebozoa sequence in an otherwise all fungal clade); 2) non-recipient eukaryotic sequences in trees with at least five genera of recipient eukaryotes; 3) sequences in trees with fewer than five genera of recipient eukaryotes; and 4) sequences from trees containing only anaerobes that are from multiple major clades of eukaryotes. The resulting collection of transcriptome and genome sequences were compared against the “nr” database using the “qblast” function in the Biopython library[112], and only sequences with robust hits to eukaryotes in the “nr” database (i.e. to taxa not included in PhyloToL) were retained (S10 Table).

We further inspected all transcriptome sequences that fell into any of the four categories and removed those that lay outside either the distribution of GC3s plotted against the ENc, or a correspondence analysis plot of the RSCU of sequences from 408 conserved gene trees (Mahalanobis distance; p-value < .001). We kept sequences that robustly hit multiple closely-related species in the nr database, and removed those that did not. We also removed some clades of very closely-related samples for which we had large numbers of transcriptomes (e.g. multiple individuals of the genus *Hyalosphenia*) if they did not hit other closely-related species and if the inferred LGT tree had a topology within eukaryotes inconsistent with vertical inheritance (i.e. interdigitation of species between genera; S10 Table). In other words, we took considerable care to rule out contamination and misidentification as we made inferences about LGTs from taxa represented only by transcriptomic data.

We took a separate approach for the curation of sequences from taxa with a whole genome assembly available (S9 Table) by evaluating whether these sequences mapped to genuine eukaryotic contigs or instead represented contaminating bacterial sequences (S9 Table). We removed all sequences that mapped to contigs shorter than 10 kb, as well as those on contigs containing no other annotated protein-coding regions (CDS). For genomic sequences on contigs longer than 10 kb that hit no closely-related eukaryotes by BLASTp against the “nr” database, we analyzed nearby CDSs and CDSs at the ends of the contig using BLASTp; here our goal was to look for bacterial material in these assemblies. If very few of these robustly hit closely-related eukaryotes and/or many robustly hit only prokaryotes, we removed the sequences. Aberrance in compositional bias was not used as a criterion in determining robustness of genomic sequences.

### Additional gene-tree curation

As a final curation step, we looked for cases in which GF designations in OrthoMCL split homologs into multiple gene trees, leading to an overestimation of LGTs. To this end, we choose representative sequences from each candidate LTG tree (i.e. sequences representing clusters of ≥75% identity generated using the VSEARCH [113]—cluster_smallmem command-line tool) and used the BLASTp tool in the BLAST+ executables package [114] to identify homologs among our original 13,630 GFs. We combined GFs for all sequences of candidate LTGs that hit an alternative gene family(s) with an e-value of an order of magnitude at least half of that of its best hit, and assessed homology using Guidance as described above. We manually inspected gene trees generated for all resulting alignments and removed eight candidate LGTs in which “recipient” eukaryotes interdigitated with other eukaryotes introduced by the alternative GF. We combined an additional 17 candidate LTGs either with other LTGs or with GFs not initially selected by our methods. Twenty-five gene families combined with alternative gene trees exhibited near-reciprocal monophyly of GFs (i.e. represented ancient gene duplication), and therefore were not changed except for the removal of contaminating sequences that interdigitated among other eukaryotes in the alternative gene family.

We evaluated the few candidate LTG trees with polyphyletic eukaryotes by AU testing, constraining topologies to have eukaryotic monophyly; putative EGTs were not tested except for those containing non-photosynthetic taxa not sister to the putative recipient clade. Constraint-tree construction and AU testing was conducted using IQ-Tree [115] through the CIPRES Science Gateway [116] REST API. The final trees available in the supplementary materials were constructed using IQ-Tree version 2.1.2 through the CIPRES Science Gateway REST API using the LG model, gamma site rate distribution (-m LG+G) and 1,000 ultrafast bootstraps (-bb 1000), and we include the most likely constrained trees for cases in which eukaryotic monophyly was accepted.

### Functional analysis

To assess the function of LGTs, we analyzed Gene Ontology [38] (GO) terms returned for each sequence using the EggNOG-mapper tool [117], implementing the Diamond model [118] under default parameters. We obtained additional GO terms using the InterPro2GO online database [119], accessed in April 2021, and PFam domains [39] identified by HMMer [120] for each sequence with a maximum domain overlap of 5 amino acids and an e-value of 1x10^-5^. GO terms were slimmed using the generic GO-Slim database as accessed on the GO website (http://current.geneontology.org/ontology/subsets/goslim_generic.obo). We mapped functional descriptions onto single gene trees and manually evaluated the results as we finalized parameters. We summarized functional categories (Fig 3A and S6–S8 Tables) by analyzing the overlap of GO term presence in GFs in each recipient category using UpSet plots (Fig 3B; created using the UpSetR package [121]; each combination of GO terms was manually assigned to each broad category) and custom Python scripts.

### Branch length ratio calculation & comparison

To assess the alternative hypothesis that exclusive presence in LTG trees is due to differential loss of genes that were present in the last eukaryotic common ancestor, we compared relative branch lengths between putative recipient and donor lineages in LTG trees to those in a corresponding set of highly conserved gene families sampled to mimic gene loss. The basis of this assessment is that the divergence between eukaryotes and prokaryotes in trees that mimic gene loss should be much greater compared to LTG trees, even in comparisons of highly conserved genes (i.e. our assessment is conservative). Though comparing the ratio of branch lengths within eukaryotic recipients to the last common ancestor with prokaryotes accounts for varying functional constraint between GFs, the analysis is based on the assumption of homogeneous substitution rates (i.e. constant functional constraint) across lineages within a given tree.

For all gene trees containing a single putative interdomain transfer event, we selected a set of 20 to 50 conserved trees (i.e. present in all five eukaryotic major clades, as well as in diverse prokaryotes) that contained all the eukaryotes and prokaryotes found within the LTG tree. Branch length comparisons were not conducted for LTGs with fewer than 20 matching conserved genes, except for the three intradomain transfers analyzed and marked in Fig 4C. We subsampled the conserved trees to match the taxonomic distribution of both eukaryotes and prokaryotes in the LTG tree, plus or minus one taxon (mimicking the gene loss necessary to explain the taxonomic distribution). In cases where there are multiple recipient clades within an LTG tree and eukaryotic monophyly could be rejected by AU testing, we either selected the eukaryotes from the largest clade (when only one recipient major clade present) or we tested both clades (when multiple major eukaryotic groups present). When paralogs were present in the eukaryotic clade in LTG trees, paralogous sequences on branches closest to the eukaryotic root were selected and all others were removed when rebuilding the tree. In VTG trees, paralogous sequences on branches furthest to the eukaryotic root were selected and all others were removed when rebuilding the tree. This served to mitigate any potential heterogeneity in substitution rates and biased towards the null hypothesis of equal branch length ratios in LTG and VTG trees exhibiting gene loss. For the seven gene families where anaerobes of two different eukaryotic major clades are sister to each other in an LGT tree, we focused only on assessment of the intradomain LGT by sampling conserved gene trees to match the taxonomic distribution of the eukaryotic clade under consideration only (i.e. no prokaryotes were included in the subsampled tree).

Examination of the average branch length within eukaryotic clades in LGT trees as compared to subsampled vertical trees revealed potential discrepancies in substitution rate, inconsistent with the assumption of homogenous substitutions (S1–S3 Figs). This measure in LTG trees frequently fell above the distribution of that in the corresponding VTG trees, hence lowering the relative distance between eukaryotes and prokaryotes. This observation holds for both LGT and EGT GFs (S1–S3 Figs), and is consistent with both differential loss of rapidly evolving genes and gene transfer, as accelerated evolutionary rates of a gene following transfer is possible. Analysis of the absolute distance between eukaryotes and prokaryotes revealed strong bias of LGT trees to lie below their corresponding VTG distributions, which is inconsistent with gene loss and consistent with LGT (S1–S3 Figs).

For all relative branch length comparisons, we used MAFFT version 7.407 to align the amino-acid sequences of all LTG and subsampled VTG trees. We then constructed single-gene trees for both VTGs and LTGs using IQ-Tree version 2.12 through the CIPRES Science Gateway REST API, using the LG model and gamma site rate distribution (-m LG+G). To calculate the relative branch lengths, each resulting tree was rooted on the eukaryotic clade (trees that mimicked gene loss where the eukaryotes were non-monophyletic were not considered) and the distance from the base of the eukaryotic clade to the base of the prokaryotes was divided by the average branch length within the recipient eukaryote clade.

In the eukaryote-only trees generated to emulate intradomain LGT among anaerobic eukaryotes, the distance between the clades of each eukaryotic major group was compared to average branch lengths within them. Each LTG tree ratio was then compared to its distribution of conserved GFs (Fig 4C and S11 and S12 Tables). Conserved gene families were not included in the control set if AU testing rejected reciprocal monophyly of the two eukaryotic groups.

## Supporting information

S1 Table List of all taxa used in the study that have putative LTGs. Columns include: major clade (Amoebozoa (Am), Archaea (Za), Archaeplastida (Pl), Bacteria (Ba), Excavata (Ex), Opisthokonta (Op), SAR (Stramenopila, Alveolata, Rhizaria), and orphan lineages (EE)); taxon code and name, the number of putative LTGs, data type (genomic, EST or Illumina), data source (i.e. the database, institution or project from which the data was accessed), the accession numbers for the data used where applicable and taxonomy as designated by NCBI.
(XLSX)

S2 Table All of the GFs inferred to be transferred into non-anaerobic organisms and their putative recipient eukaryotic clades. Gene families with two or more OG5 numbers from OrthoMCL (column A) were determined to be homologs as described in the methods section. LTGs found in only one recipient clade (column B) and that returned sufficient functional information (Gene Ontology terms; column C) are assigned broad functional categories as illustrated in Figs 2 and S1. Additional functional information is given in the form of EC numbers, Pfam IDs and PFam domain names as returned by the EggNOG mapper tool and through a search with HMMer, respectively.
(XLSX)

S3 Table All of the putative LTGs found exclusively or nearly-exclusively in anaerobic eukaryotes. The predicted function for each gene family is reported if there is consensus among the annotations of the genomic eukaryotic sequences in OrthoMCL version 5.0. We also list putative recipients and donors, the latter defined as the most coherent/broadest grouping of prokaryotes with the LTG. For each GF, we include EC numbers, PFam IDs, and PFam domain names as returned by the EggNOG mapper tool and through a search with HMMer, respectively.
(XLSX)

S4 Table Presence/absence data for all putative LTGs in every taxon listed in S1 Table. Presence of an LTG is denoted with a “1” regardless of the number of copies of the gene found in the genome (i.e. paralogs), and absence is denoted with a “0”.
(XLSX)

S5 Table Presence/absence data for conserved GFs in every taxon listed in S1 Table. Presence of a GF is denoted with a “1” regardless of the number of copies of the gene found in the genome (i.e. paralogs), and absence is denoted with a “0”. We use these conserved GFs to assess the quality of transcriptomic data, and for subsampling to generate trees that mimic gene loss in branch-length comparisons (S11 and S12 Tables).
(XLSX)

S6 Table The presence of each Gene Ontology (GO) term as returned by EggNOG and PFam across recipient groups (All Go Terms; columns B-F), those returned just by EggNog (G-K) and by Pfam only (L-P); GO terms were slimmed using the generic GO-Slim database as accessed on the GO website (http://current.geneontology.org/ontology/subsets/goslim_generic.obo) in April of 2021 and then counted. We also give the number of GFs with eukaryotic sequences that return each GO term through either source.
(XLSX)

S7 Table The presence of all un-”slimmed” Gene Ontology (GO) terms as returned by EggNOG and PFam across all putative LTGs unique to Opisthokonta or photosynthetic eukaryotes. A “1” denotes presence regardless of the number of eukaryotic sequences that returned the GO term, and a “0” denotes absence in eukaryotes.
(XLSX)

S8 Table The presence of all PFam domains as returned by HMMer across all putative LTGs. A “1” denotes presence regardless of the number of eukaryotic sequences that returned the domain, and a “0” denotes absence in eukaryotes.
(XLSX)

S9 Table All curated genomic sequences and the criteria used for determining their inclusion or exclusion. For each species, we provide the unique sequence identifier (column D), the reason for inclusion or exclusion (columns H-J), the sequence length, the length of the contig on which the putative LTG is placed (column H), the accession of the genomic data, and whether the sequence hit closely-related species or multiple other CDS on the contig hit closely-related species (columns I,J). Any notable literature pertaining to the sequence or GF is noted in the “other” column.
(XLSX)

S10 Table All curated transcriptomic sequences and the criteria used for determining their inclusion or exclusion. For each species, we provide the unique sequence identifier (column D), the reason for inclusion or exclusion (columns G-J) and whether the sequence hit closely-related species.
(XLSX)

S11 Table The branch-length distributions for all putative interdomain LGTs. For each LGT (column A) we provide distance for all comparison trees (Column B) for both LGT and VGT trees (column C). All subsampled VTG measurements are given for each corresponding LTG, and the recipient category of the LTGs (and their corresponding subsampled VTGs) match those in figures (Figs 2E and 3C and S1–S3).
(XLSX)

S12 Table The branch-length distributions for all putative intradomain LGTs. For each LGT (column A) we provide distance for all comparison trees (Column B) for both LGT and VGT trees (column C). All subsampled VTG measurements are given for each corresponding LTG; these LTGs match those at the bottom of Fig 3C.
(XLSX)

S1 Fig Measurements of relative branch length in twelve EGT trees containing only archaeplastida are consistent with those in non-photosynthetic recipient groups (Fig 2C). (A) For the majority of the EGTs, the ratio of the average branch length within the putative recipient eukaryote clade (EE) to the distance between the eukaryote clade and the prokaryotes (EP; red dots) lies outside estimates of their corresponding subsampled-VTG distributions (box plots; S11 Table). Either most or all of the prokaryotes in these trees are Cyanobacteria, consistent with plastid ancestry of these GFs. (B) Average branch length within the eukaryotic clade of the same 12 LTGs (red dots, GFs in the same order) are variable relative to their corresponding subsampled-VTG distributions (box plots), consistent with variable functional constraints on these GFs following transfer. (C) For the majority of GFs, distance between the eukaryote and prokaryote clades in the same 12 LTGs (red dots) are shorter than their corresponding subsampled-VTG distributions (box plots).
(TIFF)

S2 Fig Measurements of relative branch length in 21 LGT trees containing only Amoebozoa (blue boxes, 7 GFs), Excavata (yellow boxes, 8 GFs) and SAR (red boxes, 6 GFs). (A) In the majority of cases, the ratio between the average branch length within the putative recipient eukaryote clade (EE) and the distance (branch length) between the eukaryote clade and the prokaryotes (EP; red dots) are outside the range of their corresponding subsampled-VTG distributions (box plots). (B) The average branch length within the eukaryotic clade of the same LTGs (in the same order, red dots) are variable compared to their corresponding subsampled-VTG distributions (box plots). (C) The distance between the eukaryotic and prokaryotic clades in the same LTGs (red dots) tend to be smaller than in the same subsampled-VTG distributions.
(TIFF)

S3 Fig The average branch length within eukaryotic clades and between eukaryotes and prokaryotes of LTGs in Opisthokonta following order of GFs in Fig 2C. (A) The average branch length within clades of Opisthokonta in LTG trees tends to be longer than in their corresponding subsampled-VTG distributions. (B) The distance (branch length) between the Opisthokont clade and the prokaryote clade in LTG trees tends to be shorter than in their corresponding subsampled-VTG distributions.
(TIFF)

S4 Fig The distributions of possible donor lineages in LTG trees, with the caveat that both gene loss and prokaryote-prokaryote LGT after interdomain gene transfer events can obscure inferences. For each archaeal (top) and bacterial (bottom) clade that appeared abundantly in LTG trees unique to anaerobes (left panel), photosynthetic eukaryotes (central panel), or fungi (right panel), we measured the proportion of the taxa in that clade in the PhyloToL databases that appear in each tree (X-axis). For putative transfer events into anaerobic eukaryotes, there is greater representation of Methanobacteria, Thermotogae, Fusobacteria, and the low numbers of Proteobacteria and Cyanobacteria; Cyanobacteria are overrepresented in the EGT trees, as expected for genes involved in photosynthesis; and with LTGs in fungi there are more Alpha-proteobacteria, gamma-proteobacteria and Actinobacteria.
(TIFF)

S5 Fig Examples of data curation, including assessment of contamination by plastid-encoded (a) and other sequences in transcriptomes (b). (A) GC content at third-position four-fold degenerate sites plotted against the effective number of codons (ENc) shows that the majority of sequences in the diatom *Extubocellulus spinifer* in EGT trees (green) match patterns of sequences from conserved gene trees (orange); significantly outlying points (red; Mahalanobis distance; p < .001) may be plastid encoded and these GFs were removed. Inset is a violin plot of the GC content of conserved sequences. (B) The same set of sequences plotted in a correspondence analysis showing that relative synonymous codon usage is significantly different for the same GFs (red box; assessed by Mahalanobis distance; p < .001). (C) We also assessed the composition of transcriptomic sequences that appeared in clades lacking robust taxonomic representation (red lineages in tree; in this case, the stramenopile *Devalopayella elegans*) and removed those with compositional patterns distinct from highly conserved GFs (orange). Points in gray belong to the highly-conserved gene families, but returned top BLAST hits to bacteria with an e-value 10^3^ times lower than to eukaryotes.
(TIFF)

S6 Fig An example EGT tree. Genes subject to EGT were identified by exclusive presence in photosynthetic eukaryotes, including the Archaeplastida that acquired their plastid from a cyanobacterial ancestor and lineages that acquired plastids secondarily (e.g. photosynthetic members of SAR). Apicomplexa and Perkinsozoa occasionally appear in EGT trees, consistent with photosynthetic ancestry in these non-photosynthetic organisms.
(TIFF)

S7 Fig The distribution of prokaryotic presence in putative LGT & EGT trees. (A) the number of gene trees in which each pair of bacterial/archaeal clades co-occur. (B) the number of gene families in which each prokaryotic clade is present (bars are in the same order as in (A)). (C) The number of prokaryotic clades in each gene tree based on the data in S4 Table; the “Cyano only” label highlights trees with a single bacterial clade, all but one of which are EGTs and contain only cyanobacteria.
(TIFF)

S8 Fig A decarboxylase showing putative LGT from fungi to *Phytophthora ramorum*. Fungi are in purple, *P*. *ramorum* in red. All other tips are bacteria or archaea.
(TIFF)

S9 Fig Distribution of pairwise identities between prokaryotes and eukaryotes among LGTs (blue), EGTs (green) and the VGT trees that we generated to mimic gene loss (red), the latter of which were sampled from conserved gene families. Across all gene trees, we calculated the pairwise identities between each eukaryotic and prokaryotic sequence after aligning each pair of sequences separately. Few pairs exceed 70% identity (vertical dashed line), an observation consistent with the “70% rule” defined by Ku and Martin (2017), who argued that interdomain comparisons with >70% identity are likely contaminants. The right skew of the VTG distribution is consistent with the conservative nature of these gene families, which we selected based on their wide distribution among eukaryotes and prokaryotes.
(TIFF)
