# Supplementary figures and images for: Old genes in new places: A taxon-rich analysis of interdomain lateral gene transfer events
Source: PLoS Genet. 2022 Jun 22;18(6):e1010239. doi: 10.1371/journal.pgen.1010239 (PMC9255765; doi:10.1371/journal.pgen.1010239)

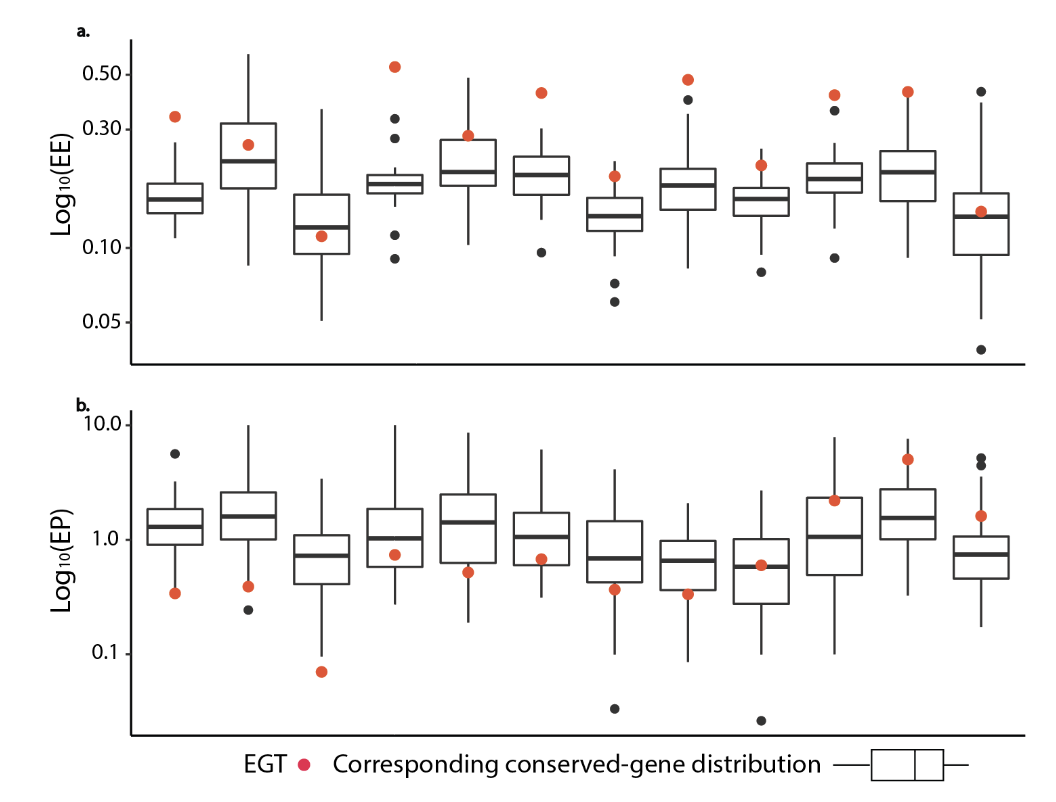

Supplement: S1 Fig — (A) For the majority of the EGTs, the ratio of the average branch length within the putative recipient eukaryote clade (EE) to the distance between the eukaryote clade and the prokaryotes (EP; red dots) lies outside estimates of their corresponding subsampled-VTG distributions (box plots; S11 Table). Either most or all of the prokaryotes in these trees are Cyanobacteria, consistent with plastid ancestry of these GFs. (B) Average branch length within the eukaryotic clade of the same 12 LTGs (red dots, GFs in the same order) are variable relative to their corresponding subsampled-VTG distributions (box plots), consistent with variable functional constraints on these GFs following transfer. (C) For the majority of GFs, distance between the eukaryote and prokaryote clades in the same 12 LTGs (red dots) are shorter than their corresponding subsampled-VTG distributions (box plots). (TIFF) [file pgen.1010239.s013.tiff]

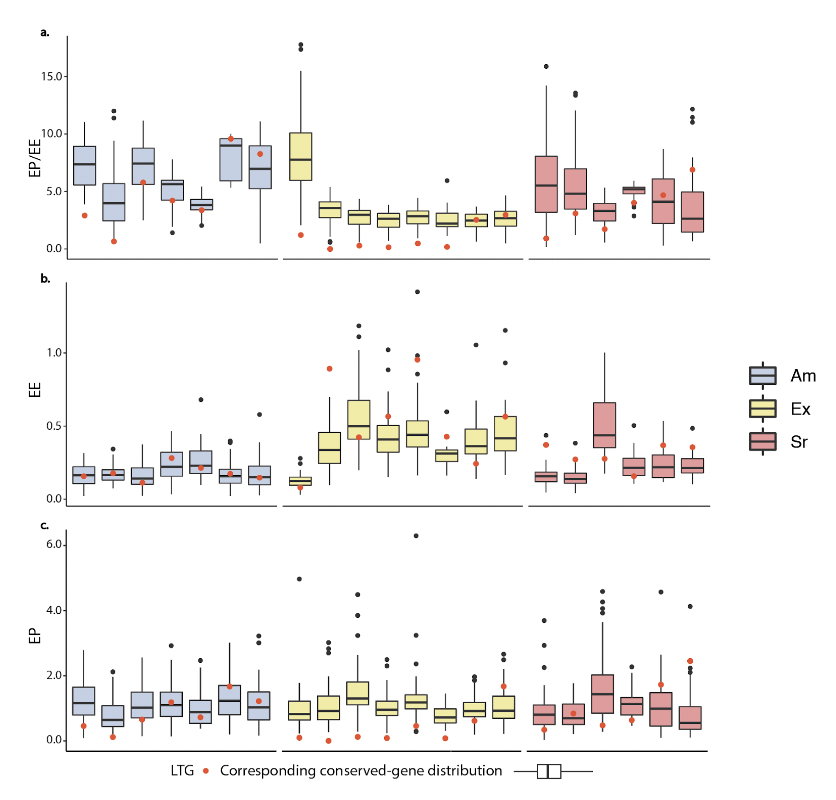

Supplement: S2 Fig — (A) In the majority of cases, the ratio between the average branch length within the putative recipient eukaryote clade (EE) and the distance (branch length) between the eukaryote clade and the prokaryotes (EP; red dots) are outside the range of their corresponding subsampled-VTG distributions (box plots). (B) The average branch length within the eukaryotic clade of the same LTGs (in the same order, red dots) are variable compared to their corresponding subsampled-VTG distributions (box plots). (C) The distance between the eukaryotic and prokaryotic clades in the same LTGs (red dots) tend to be smaller than in the same subsampled-VTG distributions. (TIFF) [file pgen.1010239.s014.tiff]

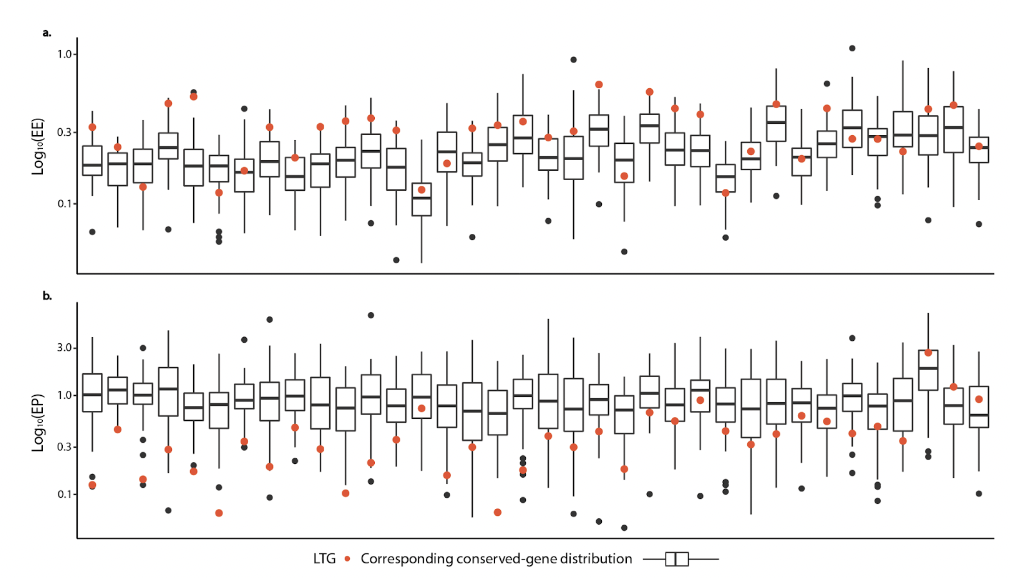

Supplement: S3 Fig — (A) The average branch length within clades of Opisthokonta in LTG trees tends to be longer than in their corresponding subsampled-VTG distributions. (B) The distance (branch length) between the Opisthokont clade and the prokaryote clade in LTG trees tends to be shorter than in their corresponding subsampled-VTG distributions. (TIFF) [file pgen.1010239.s015.tiff]

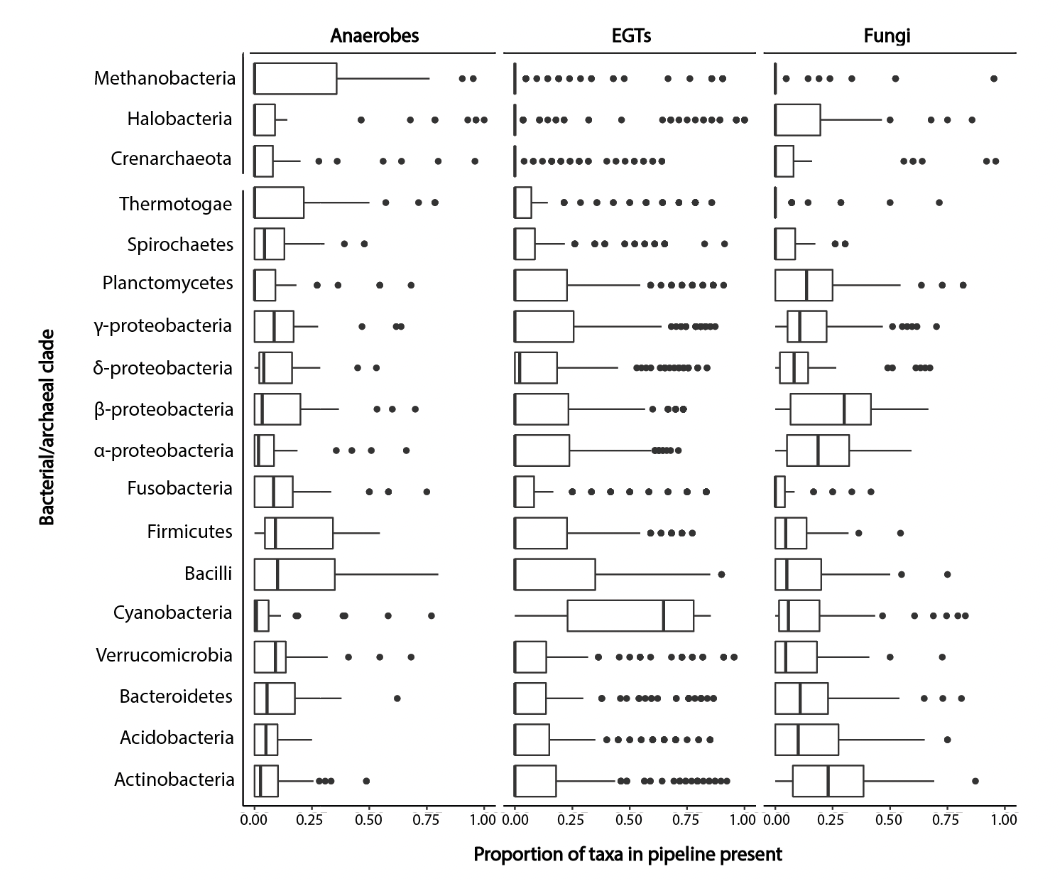

Supplement: S4 Fig — For each archaeal (top) and bacterial (bottom) clade that appeared abundantly in LTG trees unique to anaerobes (left panel), photosynthetic eukaryotes (central panel), or fungi (right panel), we measured the proportion of the taxa in that clade in the PhyloToL databases that appear in each tree (X-axis). For putative transfer events into anaerobic eukaryotes, there is greater representation of Methanobacteria, Thermotogae, Fusobacteria, and the low numbers of Proteobacteria and Cyanobacteria; Cyanobacteria are overrepresented in the EGT trees, as expected for genes involved in photosynthesis; and with LTGs in fungi there are more Alpha-proteobacteria, gamma-proteobacteria and Actinobacteria. (TIFF) [file pgen.1010239.s016.tiff]

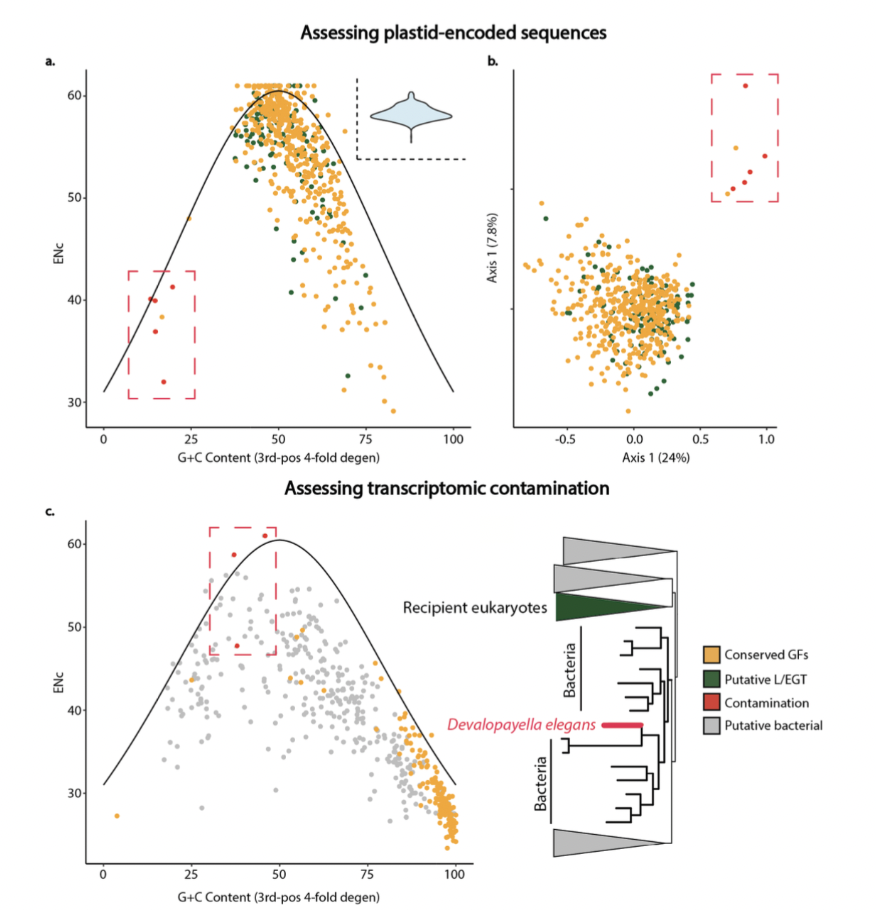

Supplement: S5 Fig — Examples of data curation, including assessment of contamination by plastid-encoded (a) and other sequences in transcriptomes (b). (A) GC content at third-position four-fold degenerate sites plotted against the effective number of codons (ENc) shows that the majority of sequences in the diatom Extubocellulus spinifer in EGT trees (green) match patterns of sequences from conserved gene trees (orange); significantly outlying points (red; Mahalanobis distance; p < .001) may be plastid encoded and these GFs were removed. Inset is a violin plot of the GC content of conserved sequences. (B) The same set of sequences plotted in a correspondence analysis showing that relative synonymous codon usage is significantly different for the same GFs (red box; assessed by Mahalanobis distance; p < .001). (C) We also assessed the composition of transcriptomic sequences that appeared in clades lacking robust taxonomic representation (red lineages in tree; in this case, the stramenopile Devalopayella elegans) and removed those with compositional patterns distinct from highly conserved GFs (orange). Points in gray belong to the highly-conserved gene families, but returned top BLAST hits to bacteria with an e-value 103 times lower than to eukaryotes. (TIFF) [file pgen.1010239.s017.tiff]

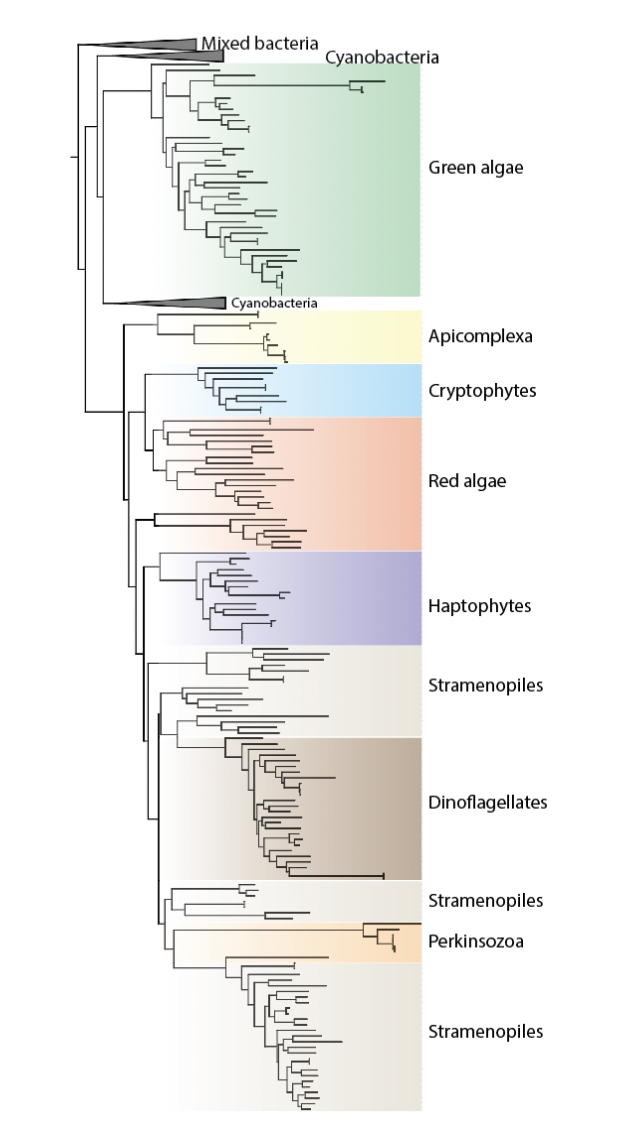

Supplement: S6 Fig — Genes subject to EGT were identified by exclusive presence in photosynthetic eukaryotes, including the Archaeplastida that acquired their plastid from a cyanobacterial ancestor and lineages that acquired plastids secondarily (e.g. photosynthetic members of SAR). Apicomplexa and Perkinsozoa occasionally appear in EGT trees, consistent with photosynthetic ancestry in these non-photosynthetic organisms. (TIFF) [file pgen.1010239.s018.tiff]

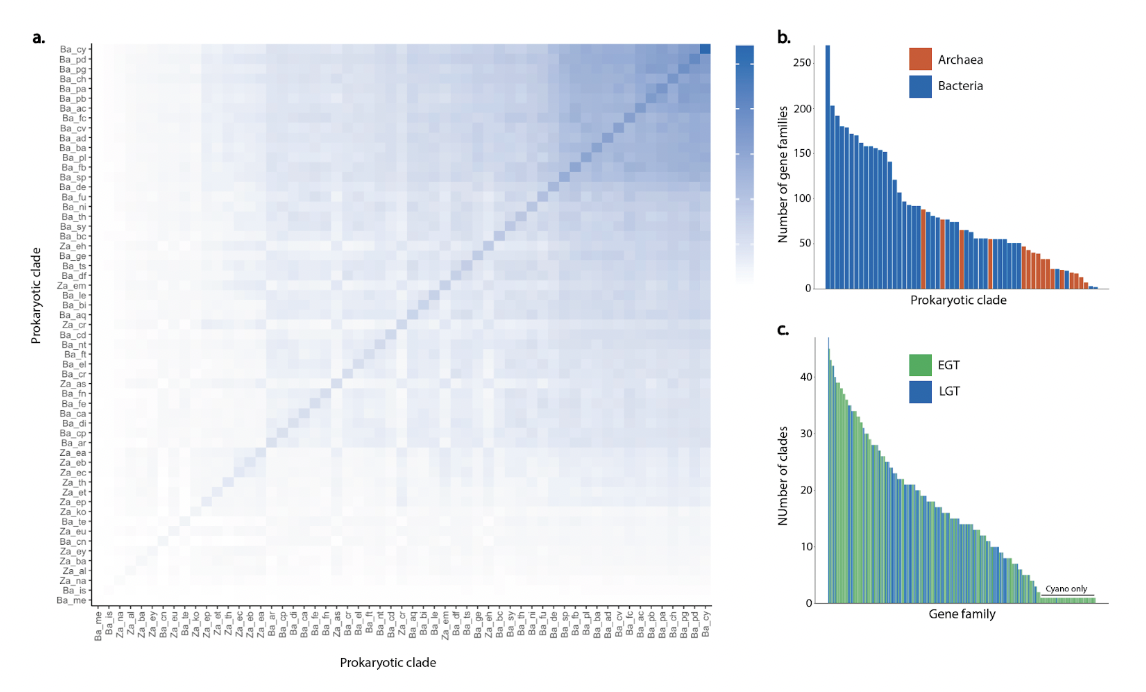

Supplement: S7 Fig — (A) the number of gene trees in which each pair of bacterial/archaeal clades co-occur. (B) the number of gene families in which each prokaryotic clade is present (bars are in the same order as in (A)). (C) The number of prokaryotic clades in each gene tree based on the data in S4 Table; the “Cyano only” label highlights trees with a single bacterial clade, all but one of which are EGTs and contain only cyanobacteria. (TIFF) [file pgen.1010239.s019.tiff]

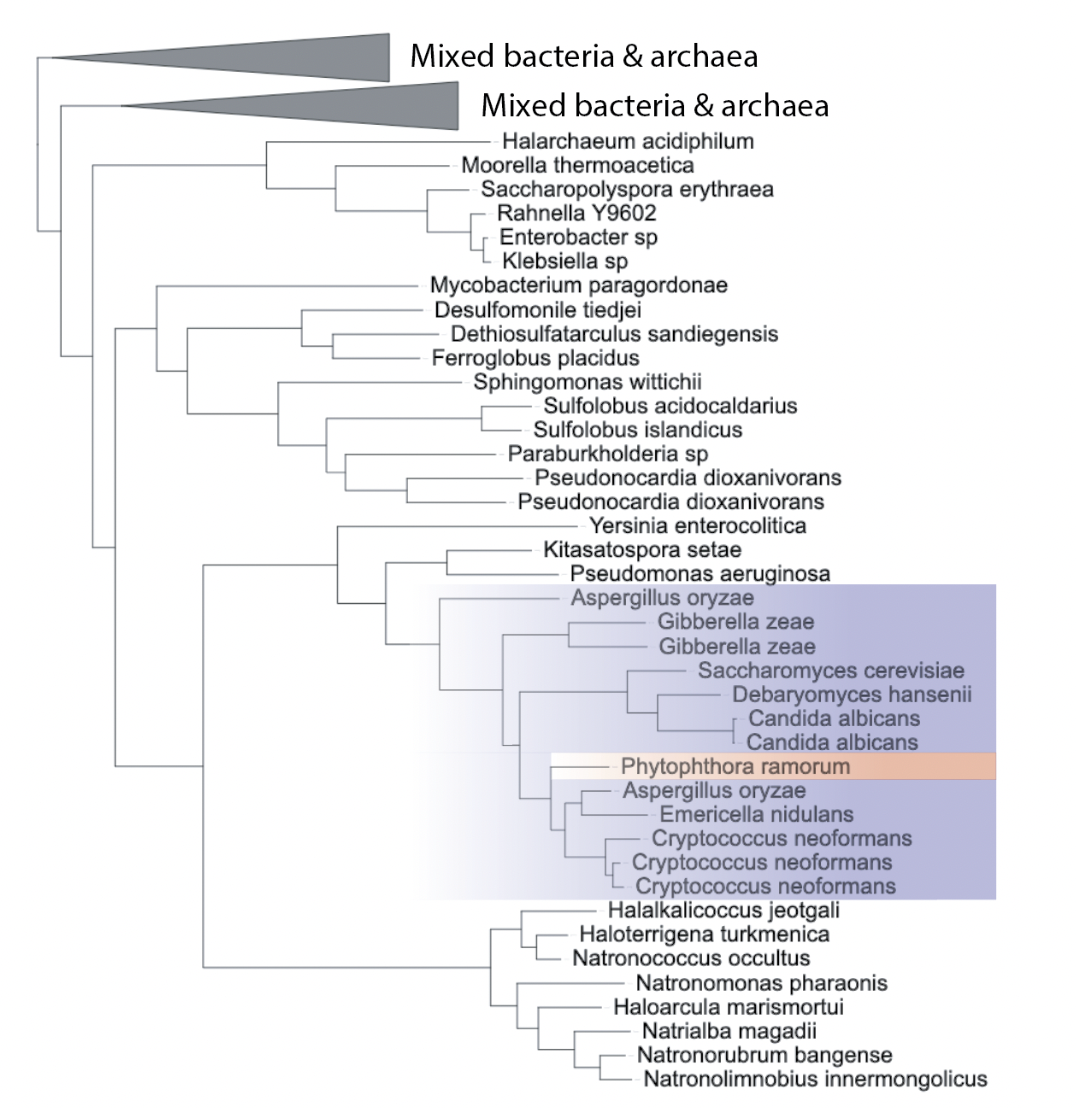

Supplement: S8 Fig — Fungi are in purple, P. ramorum in red. All other tips are bacteria or archaea. (TIFF) [file pgen.1010239.s020.tiff]

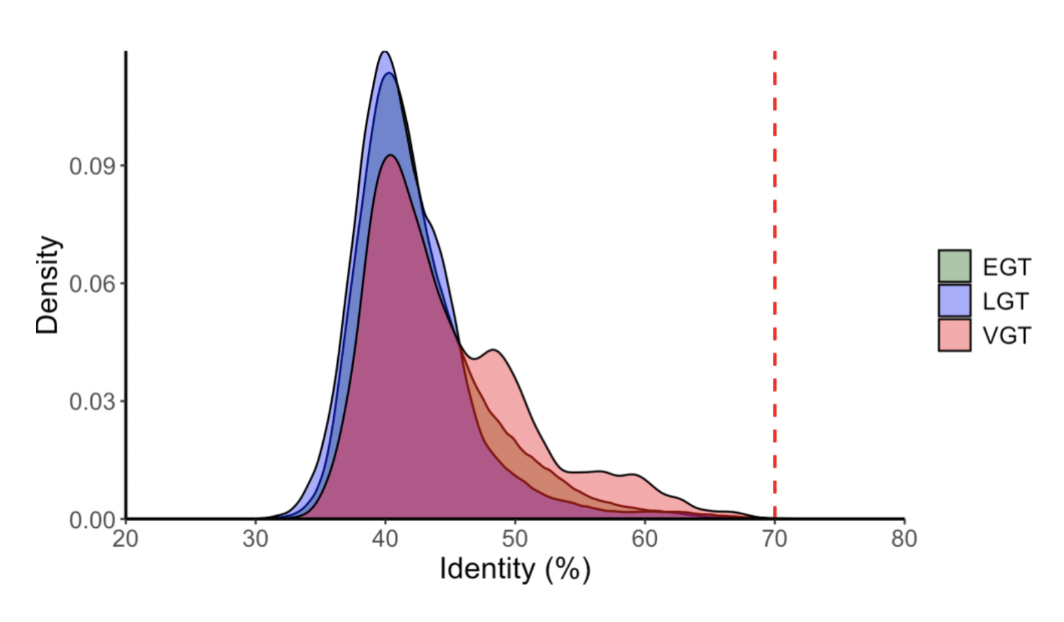

Supplement: S9 Fig — Across all gene trees, we calculated the pairwise identities between each eukaryotic and prokaryotic sequence after aligning each pair of sequences separately. Few pairs exceed 70% identity (vertical dashed line), an observation consistent with the “70% rule” defined by Ku and Martin (2017), who argued that interdomain comparisons with >70% identity are likely contaminants. The right skew of the VTG distribution is consistent with the conservative nature of these gene families, which we selected based on their wide distribution among eukaryotes and prokaryotes. (TIFF) [file pgen.1010239.s021.tiff]
